# Predicting Post-Concussion Symptom Risk in the ED

**DOI:** 10.15844/pedneurbriefs-30-3-2

**Published:** 2016-03

**Authors:** Anthony P. Kontos, Jaime McAllister-Deitrick, Alicia M. Sufrinko

**Affiliations:** 1 Department of Orthopaedic Surgery, University of Pittsburgh, Pittsburgh, PA

**Keywords:** Concussion, Postconcussion Syndrome, Mild Traumatic Brain Injury, Children, Pediatrics

## Abstract

Investigators from The Pediatric Emergency Research Canada (PERC) Concussion Team developed a clinical risk score for predicting persistent post-concussion symptoms (PPCS) at 28 days post injury in a large cohort of children initially evaluated at the emergency department (ED) within 48 hours of injury.

Investigators from The Pediatric Emergency Research Canada (PERC) Concussion Team developed a clinical risk score for predicting persistent post-concussion symptoms (PPCS) at 28 days post injury in a large cohort of children initially evaluated at the emergency department (ED) within 48 hours of injury. This prospective multicenter study first recruited a derivation cohort of 2006 patients followed by a validation cohort of 1057 patients across 9 Canadian Hospitals. Persistent post-concussion symptoms were present in 31.0% of the derivation cohort and 33.0% of the validation cohort. The initial 12 predictor PPCS risk score from the derivation sample was refined to a final risk score that incorporated 9 predictors including: age, sex, prior concussion with symptom duration of longer than 1 week, physician-diagnosed migraine history, headache, sensitivity to noise, fatigue, answering questions slowly, and abnormal tandem stance balance performance. The researchers also compared the ability of the new PPCS clinical risk score to accurately predict PPCS with physician prognosis. Treating physicians completed a standardized survey, which included the question, “How likely is this patient to develop persistent symptoms beyond 1 month?” with response options starting with 0-10% and increasing in 10% increments up to 100%. The PPCS clinical risk score was significantly better than physician prognosis for predicting PPCS at 28 days post-injury. However, the discrimination of the PPCS risk score model was moderate (AUC=0.71) and requires further refinement and validation. The authors suggest further research is needed for assessment of accuracy in an office setting and determination of clinical utility before the score is adopted in clinical practice. [1]

COMMENTARY. Given the increasing number of concussions seen in pediatric EDs over the last decade [2], concussion has become a serious public health concern [3]. Although most patients recover within 2 weeks, approximately one-third of patients will continue to experience symptoms (i.e., PPCS) and impairment for a prolonged period following a concussion. Determining which factors at the time of presentation to the ED predict PPCS is important to inform better prognosis and potential early interventions for patients. Researchers have demonstrated that certain factors (e.g., age, sex, post-traumatic migraine) may place patients at greater risk for prolonged recovery [4, 5]. Currently, there is no validated clinical algorithm for predicting PPCS in patients. Developing such an algorithm for use in the pediatric ED could substantially reduce morbidity associated with concussion and inform better follow-on care for this at-risk population.

The current study was the first to attempt to quantify risk for PPCS in a large sample of pediatric patients based on factors routinely assessed during patient evaluations in the ED. The authors utilized brief clinical assessments commonly used in the ED, and assigned risk points for each variable to assess the overall risk for PPCS in two independent samples. The resulting risk score stratifies patients into low, medium, or high risk for PPCS. Unfortunately, the current model only provided modest discrimination of risk for PPCS. Additional data on the sensitivity and specificity of the risk score with additional independent samples from other clinical settings that include concussed patients and controls are needed before the PPCS risk score can be applied clinically.
